# Supplementary material for: Oilseed rape (Brassica napus) resistance to growth of Leptosphaeria maculans in leaves of young plants contributes to quantitative resistance in stems of adult plants
Source: PLoS One. 2019 Sep 12;14(9):e0222540. doi: 10.1371/journal.pone.0222540 (PMC6742359; doi:10.1371/journal.pone.0222540)
Supplement: S2 Table — (DOCX) [file pone.0222540.s002.docx]

**S2 Table. List of eleven field experiments which produced QTL based on the phoma stem canker severity data (Kumar et al. 2018) used for comparison with QTL detected in controlled environment experiments with those for 190 DH lines from the DY population**

| **Population** | **Population type** | **No of lines** | **Year** | **Location** | **Reference** |
| --- | --- | --- | --- | --- | --- |
| ‘Darmor-bzh’ × ‘Yudal’ (DY) | DH | 154 | 1995 | Le Rheu, INRA, France | Pilet et al. 1998 |
|  |  | 154 | 1996 | Le Rheu, INRA, France | Pilet et al. 1998 |
|  |  | 275 | 2007 | Le Rheu, INRA, France | Jestin et al. 2012 |
|  |  | 119 | 2008 | Rothamsted, UK | Huang et al. 2016 |
|  |  | 119 | 2009 | Rothamsted, UK | Huang et al. 2016 |
|  |  | 260 | 2011 | Le Rheu, INRA, France | Fopa-Fomeiu et al. 2015 |
|  |  | 271 | 2012 | Le Rheu, INRA, France | Fopa-Fomeiu et al. 2015 |
| ‘Darmor’ × ‘Bristol’ (DB) | F2:3 | 112 | 2008 | Le Rheu, INRA, France | Jestin et al. 2015 |
|  |  | 112 | 2010 | Le Rheu, INRA, France | Jestin et al. 2015 |
| ‘Darmor’× ‘Samoura’ (DS) | DH | 116 | 1998 | Le Rheu, INRA, France | Pilet et al. 1998 |
|  |  | 116 | 1999 | Le Rheu, INRA, France | Pilet et al. 1998 |
