# Supplementary material for: Oilseed rape (Brassica napus) resistance to growth of Leptosphaeria maculans in leaves of young plants contributes to quantitative resistance in stems of adult plants
Source: PLoS One. 2019 Sep 12;14(9):e0222540. doi: 10.1371/journal.pone.0222540 (PMC6742359; doi:10.1371/journal.pone.0222540)
Supplement: S4 Table — (DOCX) [file pone.0222540.s004.docx]

**S4 Table. Linkage groups (LG) where QTL for resistance to *Leptosphaeria maculans* detected in controlled environment experiments with QTL detected in winter oilseed rape field experiments with the *Brassica napus* DY (‘Darmor-*bzh*’ x ‘Yudal’), DB (‘Darmor’ x ‘Bristol’ F_2:3_) or DS (‘Darmor’ x ‘Samourai’ DH) population**

| **Expt-trait ^a^** | **LG ^b^** | **Locus ^c^** | **Position**  **(cM) ^c^** | **Support interval (cM)** | **Effect^d^** | **LOD^e^** | ***R*^2^ (%)^f^** |
| --- | --- | --- | --- | --- | --- | --- | --- |
| DY mapping population | | |  |  |  |  |  |
| 2012-DI | A02 | BS009793 | 65.1 | 59.5-81.4 | 0.53 | 4.92 | 3.7 |
| BLUP-DI | A02 | Bn-A02-p10591779 | 61.7 | 60.3-68.5 | 0.30 | 8.38 | 3.5 |
| BLUP-DI | A03 | Bn-A03-p22976259 | 129.2 | 126.9-132.8 | -0.20 | 9.87 | 4.1 |
| 2007-DI | A03 | Bn-A03-p22816593 | 127.9 | 124.9-141.5 | -0.27 | 3.94 | 2.6 |
| 2009-DI | C01 | Bn-C1-p208224 | 0 | 0-4.5 | -0.40 | 3.44 | 5.5 |
| BLUP-DI | C09 | cC09.loc111 | 110.1 | 110-116.6 | 0.32 | 12.55 | 5.4 |
| 1995-DI | C09 | cC09.loc111 | 114.9 | 110.4-119.1 | 0.29 | 5.69 | 10.8 |
| 1996-DI | C09 | BS006734 | 119.1 | 110.4-119.1 | 0.34 | 5.79 | 5.8 |
| 2008-DI | C09 | cC09.loc111 | 110.0 | 105.6-116.6 | 0.34 | 4.26 | 12.1 |
| 2009-DI | C09 | scaffoldv4_336_615476 | 107.6 | 102.9-116.6 | 0.60 | 6.76 | 11.5 |
| DB mapping population | | |  |  |  |  |  |
| 2008-DI | A03 | scaffoldv4_453_51565 | 124.5 | 123.3-125.3 | -0.14 | 9.31 | 6.7 |
| BLUP-DI | A04 | cA04.loc23 | 23.0 | 20.7-23.6 | 0.12 | 13.88 | 3.2 |
| 2010-DI | A10 | Scaffoldv4_256_298430 | 3.7 | 1.4-4.2 | 0.16 | 15.15 | 8.2 |
| 2008-DI | C01 | cC01.loc61 | 61.0 | 59.6-62.3 | -0.12 | 11.02 | 8.2 |
| BLUP-DI | C01 | cC01.loc62 | 62.0 | 59.8-62.6 | -0.11 | 10.31 | 2.2 |
| BLUP-DI | C09 | cC09.loc16 | 16.0 | 11.0-17.6 | -0.29 | 33.16 | 12.2 |
| 2010-DI | C09 | Scaffoldv4_282_383090 | 60.1 | 55.2-60.6 | -0.21 | 14.16 | 5.8 |
| DS mapping population | | |  |  |  |  |  |
| 1998-DI | A02 | cA02.loc9 | 8.0 | 0-18.1 | 0.24 | 4.83 | 12.1 |
| BLUP-DI | A02a | cA02.loc3 | 3.0 | 0-13.4 | 0.13 | 4.02 | 10.4 |
| BLUP-DI | A02b | Bn-A02-p26435743 | 74.5 | 52.4-75.3 | 0.12 | 3.67 | 9.4 |

^a^Expt-trait, the years 1995 to 2012 are the years when the field experiments were assessed for phoma stem canker severity, BLUP (best linear unbiased predictions) - estimation of combined data for seven years; for traits, DI – disease severity index.

^b^LG, the linkage groups, are named according to *Brassica napus* A01–A10 and C01–C09 designations by the Multinational *Brassica* Genome Project Steering Committee.

(<http://www.brassica.info/information/lg_assigments.htm>)

^c^The marker closest to the position of maximum effect of the QTL.

^d^The additive effect.

^e^Test statistic value for QTL, logarithm of the odds.

^f^Proportion (%) of the phenotypic variation explained by the QTL.
