# Supplementary material for: Oilseed rape (Brassica napus) resistance to growth of Leptosphaeria maculans in leaves of young plants contributes to quantitative resistance in stems of adult plants
Source: PLoS One. 2019 Sep 12;14(9):e0222540. doi: 10.1371/journal.pone.0222540 (PMC6742359; doi:10.1371/journal.pone.0222540)
Supplement: S1 Fig — Correlations between phoma stem canker disease index in winter oilseed rape field experiments in 1995, 1996, 2007, 2008, 2009, 2011 and 2012 with doubled haploid (DH) lines from the Brassica napus DY (‘Darmor-bzh’ x ‘Yudal’) mapping population. For details of these field experiments see S2 Table. (DOCX) [file pone.0222540.s005.docx]

**S1 Fig. Correlations between disease severity index in different field experiments.** Correlations between phoma stem canker disease index in winter oilseed rape field experiments in 1995, 1996, 2007, 2008, 2009, 2011 and 2012 with doubled haploid (DH) lines from the *Brassica napus* DY (‘Darmor-*bzh*’ x ‘Yudal’) mapping population. For details of these field experiments see S2 Table.
