# Supplementary material for: Oilseed rape (Brassica napus) resistance to growth of Leptosphaeria maculans in leaves of young plants contributes to quantitative resistance in stems of adult plants
Source: PLoS One. 2019 Sep 12;14(9):e0222540. doi: 10.1371/journal.pone.0222540 (PMC6742359; doi:10.1371/journal.pone.0222540)
Supplement: S2 Fig — Comparison of the physical position (in Mb) of QTL on A03, A10 and C09 for resistance against Leptosphaeria maculans detected in controlled environment experiments (CE) in young plants with QTL detected in field experiments in adult plants. CE experiments were done with 190 lines from the Brassica napus DY (‘Darmor-bzh’ x ‘Yudal’ DH) population. Field experiments were done with the DY population in 1995, 1996, 2007, 2008, 2009, 2011 and 2012, with the DB (‘Darmor’ x ‘Bristol’ F2:3) population in 2008 and 2010 and with the DS (‘Darmor’ x ‘Samourai’ DH) population in 1998 and 1999 (for details see S1 & S2 Tables). QTL from CE experiments are in blue; QTL from individual year in field experiments are in pink; QTL from combined years of field experiments using BLUP (best linear unbiased predictions) estimation are in orange. (DOCX) [file pone.0222540.s006.docx]

**S2 Fig.** **Comparison of the physical position of QTL**. Comparison of the physical position (in Mb) of QTL on A03, A10 and C09 for resistance against *Leptosphaeria maculans* detected in controlled environment experiments (CE) in young plants with QTL detected in field experiments in adult plants. CE experiments were done with 190 lines from the *Brassica napus* DY (‘Darmor-*bzh*’ x ‘Yudal’ DH) population. Field experiments were done with the DY population in 1995, 1996, 2007, 2008, 2009, 2011 and 2012, with the DB (‘Darmor’ x ‘Bristol’ F_2:3_) population in 2008 and 2010 and with the DS (‘Darmor’ x ‘Samourai’ DH) population in 1998 and 1999 (for details see S1 & S2 Tables). QTL from CE experiments are in blue; QTL from individual year in field experiments are in pink; QTL from combined years of field experiments using BLUP (best linear unbiased predictions) estimation are in orange.
