# Supplementary material for: Oilseed rape (Brassica napus) resistance to growth of Leptosphaeria maculans in leaves of young plants contributes to quantitative resistance in stems of adult plants
Source: PLoS One. 2019 Sep 12;14(9):e0222540. doi: 10.1371/journal.pone.0222540 (PMC6742359; doi:10.1371/journal.pone.0222540)
Supplement: Attachment — Submitted filename: Responses to reviewers.docx [file pone.0222540.s007.docx]

PONE-D-19-15868
Oilseed rape (*Brassica napus*) resistance to growth of *Leptosphaeria maculans* in leaves of young plants contributes to quantitative resistance in stems of adult plants
PLOS ONE

**Response to comments from Editor**

*We would like to thank the Academic Editor Dr Harsh Raman for his time in assessing our manuscript and his advice for improving our manuscript.*

Additional Editor Comments:

Overall manuscript is well written. Please format this manuscript properly (currently in different fonts and styles etc) and address comments raised by both reviewers. Here are my further comments

L78-81: Revise sentence

Answer: Done.

L130: Insert also reference 56 (as it validates). Also, Yudal has QTL for quantitative resistance! Revise accordingly.

Answer: We don’t think it is appropriate to recite the reference 56 (in the revised version, this reference is now number 58) here because the objective here is to reference the information about the parents of the DY population when it was first developed. When the parents were chosen for the DY population, Darmor was the resistant parent (with quantitative resistance and an not effective *Rlm9*) and Yudal was the susceptible parent (those pieces of information are in references 42 and 45, so these two references were cited here); we cannot change the existing description of the parents. Although QTL contributed by Yudal were detected (on C01) in both field experiments and in controlled environment experiments in this study, it is not appropriate to put the results into the ‘Methods and Materials’, because you don’t know the results before you do the experiments. We have revised the sentence to reflect what has been published about Yudal.

L172, L274: delete nine lines. It is written in the first line

Answer: Done.

L188, 323: format headings, use the same font, delete 190 lines

Answer: We think it is better to keep the ‘190 lines’ for the level 2 heading in L188 (now L191). We deleted the ‘190 lines’ in L323 (now L338).

L197-198: You may delete, 'therefore, the condia...........inoculum".

Answer: We think it is better to leave this sentence, because this will allow the readers to understand why we used the conidial suspension of the isolate ME24.

L328: The continuous distributions?

Answer: The sentence has been revised.

L332: broad sense heritability?

Answer: The sentence has been revised.

Figure 3: Rearrange the order as A, B and C

Answer: We did not notice that the order A, B and C changed when we converted figure to a tiff file; this has now been corrected.

Figure 4; Provide high resolution image

Answer: Done.

Table 3: which version of the reference genome was used. Please describe in methods or in this Table. Physical interval in bp?

Answer: Information of the reference genome and description of the methods have been added.

Yes, the physical interval is in bp. This has been changed in Table 3.

Table 4: Please provide physical interval here
Answer: Done.

**Response to Reviewer's to Questions**

*We would like to thank the two reviewers for their time in assessing our manuscript and their advice for improving our manuscript*.

**Comments to the Author**
1. Is the manuscript technically sound, and do the data support the conclusions?

The manuscript must describe a technically sound piece of scientific research with data that supports the conclusions. Experiments must have been conducted rigorously, with appropriate controls, replication, and sample sizes. The conclusions must be drawn appropriately based on the data presented. 

Reviewer #1: Yes
Reviewer #2: Yes

2. Has the statistical analysis been performed appropriately and rigorously? 
Reviewer #1: Yes
Reviewer #2: Yes

3. Have the authors made all data underlying the findings in their manuscript fully available?
The [PLOS Data policy](http://www.plosone.org/static/policies.action#sharing) requires authors to make all data underlying the findings described in their manuscript fully available without restriction, with rare exception (please refer to the Data Availability Statement in the manuscript PDF file). The data should be provided as part of the manuscript or its supporting information, or deposited to a public repository. For example, in addition to summary statistics, the data points behind means, medians and variance measures should be available. If there are restrictions on publicly sharing data—e.g. participant privacy or use of data from a third party—those must be specified.

Reviewer #1: Yes
Reviewer #2: Yes

4. Is the manuscript presented in an intelligible fashion and written in standard English?

PLOS ONE does not copyedit accepted manuscripts, so the language in submitted articles must be clear, correct, and unambiguous. Any typographical or grammatical errors should be corrected at revision, so please note any specific errors here.

Reviewer #1: Yes
Reviewer #2: Yes

5. Review Comments to the Author

Please use the space provided to explain your answers to the questions above. You may also include additional comments for the author, including concerns about dual publication, research ethics, or publication ethics. (Please upload your review as an attachment if it exceeds 20,000 characters)

Reviewer #1: Manuscript title: Oilseed rape (Brassica napus) resistance to growth of Leptosphaeria maculans in leaves of young plants contributes to quantitative resistance in stems of adult plants (PONE-D-19-15868) by Huang et al.

The authors of this manuscript have attempted to identify the components of quantitative resistance to Leptosphaeria maculans (blackleg) in oilseed rape that are highly desirable in durable resistance breeding for blackleg. However, my main concern is that authors used only one isolate in their investigations, therefore, how the breeders are going to use this information when large number of pathogen races exists and that too keep on changing every year! Whether they should be screening against more prevalent but less virulent races or should they screen against less prevalent but more virulent ones?

Answer: Yes, we agree with reviewer that only one isolate was used; in controlled conditions it is difficult to use several isolates with 190 lines. However, quantitative resistance (QR) against *L. maculans* (Lm) is considered race non-specific. The novelty of this study is to investigate whether QTL for QR against Lm can be detected in young plants in controlled environments and whether these QTL are co-located with the QTL detected in adult plants in field conditions. The future work will test different isolates. This has now been discussed in the Discussion.

Overall, the manuscript is suitable for publication in Plos One with some specific points that need to be addressed as outlined below:
Authors chose nine DH lines of a (Darmor-bzh x Yudal) population based upon their crown/stem canker severity assessed in 1995, 1996 and 2007, which was similar in these three years; authors need to provide a reference for this.

Answer: The reference has been added to the text and the correlations between the three seasons have now been provided as S1 Fig.

In their current investigations, authors related the outcomes of glasshouse/controlled environment experiments with the stem canker severity assessed so long ago (1995, 1996 and 1997). My main concern is that why authors did not attempt to retest the stem canker severity under field conditions during their current investigations to validate their claims. There are significant amount of published papers demonstrating that population of L. maculans changes very rapidly, it is likely that the chosen DH lines may behave differently to the existing populations under field conditions to the populations they were exposed to about 11 to 20 years ago. Moreover, there is evidence that quantitative resistance is eroded over time.

Answer: There is evidence that populations of Lm change very rapidly to overcome *R* gene mediated resistance against Lm. However, QR against Lm is considered race non-specific and is considered durable. We have tested the QR in the DY population in seven field experiments in two different countries in Europe (see reference 46). Recently, this population was tested in Australia and stable QTL were detected (see reference 58), indicating that the QR in this population is durable, so our selection of representative DH lines based on previous field results was reliable (details of the 7 years field data for the 9 DH lines has now been provided in S1 Table).

In the methods section authors used ascospore inoculum from cultivar, ‘Courage’, need to provide more information regarding whether it was a susceptible or resistant cultivar and/ or the Rlm gene it carries.

Answer: Courage was a susceptible cultivar (AHDB resistance rating was 3 on a 1-9 scale, where 9 is resistant) and there is no information about the *Rlm* genes for this cultivar. The word ‘susceptible’ has now been added. This cultivar is not in use anymore.

It was interesting that authors used visual assessments to determine the growth of the fungus (Gt) down the petiole from the infection site. Generally, such a growth is systemic and often not visible in most instances so how confident authors are that this trait can be used as one of the criterion for determining quantitative resistance.

Answer: In our previous studies on investigating methods to measure growth of Lm from leaf lesions towards the stem (see reference 36), we used different types of inoculum and different measurement methods. We measured visible necrosis symptoms, used a GFP-tagged isolate to measure symptomless growth and used quantitative PCR to quantify the amount of Lm DNA. Different measurements led to similar conclusions (i.e. Lm grew more in susceptible DH lines than in resistant DH lines). Using GFP isolates is time-consuming and there are restrictions in experimental conditions due to biosafety regulations, using qPCR is expensive and time-consuming, measuring the visible distance grown is simple and quicker. There were visible differences between different DH lines (Fig. 2). Therefore, we are confident that the trait we measured is appropriate for determining QR.

My other concerns is why authors did not include an absolute susceptible check to compare the Gt in DH lines to that of a susceptible line. I understand they have used one susceptible DH line but including a highly susceptible check would have been much valuable.

Answer: We don’t understand what the term ‘absolute susceptible check’ means. We guess this means a very susceptible commercial cultivar. Yes, including a susceptible cultivar will provide information about the susceptibility of the cultivar in controlled conditions compared with that of susceptible DH lines. However, this information does not provide any value to farmers/breeders. We don’t think there is a need to include a susceptible cultivar because this study is to investigate QTL for resistance against Lm; there are already a wide range of differences between different DH lines within the mapping population.

It is not very clear how many plants per DH line for each replication were used in the first experiment.

Answer: For the experiments with nine DH lines, there were 12 plants for each DH line for each replicated experiment (previous Line 153-155, ‘Two experiments were done in a complete randomised block design with 12 blocks with one plant of each DH line in each block’). To make this clearer, we have now revised this sentence as ‘Two experiments were done in a complete randomised design with 12 plants for each DH line’.

In the second experiment with 190 DH lines, it appears that two plants per DH lines were used and there were three reps that equates to just six plants for each DH line in total. In my view, the experimental unit is too small to draw useful conclusions.

Answer: In controlled conditions with 190 DH lines, it is difficult to have a large number of plants for each DH line. We believe that a total of six plants for each DH line is a reasonable number because there are 12 data points for each DH line (6 plants × 2 leaves/plant). Our statistician helped us with the experimental design. The experiment was repeated once (i.e. two experiments were done with the 190 DH lines).

In addition, in the second experiment, authors maintained high humidity for 72 hours, whereas, in the first experiment, this was for 48 hours; authors need to mention the reasons for this inconsistency. This will be helpful for readers who are not familiar with the relative infection ability of ascospores vs pycnidiospores.

Answer: One sentence has now been added and a reference has been added.

In Table 1, authors need to provide the severity rating scale (for example, 0-5 or 0-4 etc.) used in 1995, 1996 and 2007 experiments in France for calculating the stem canker disease index (DI) and bit more details on how the DI was calculated.

Answer: The stem canker was assessed on a 0-9 disease score scale, the disease index was calculated using the following formula: Disease index (DI) = [(N_0_ × 0) + (N_1_ × 1) + (N_2_ × 2) + … + (N_9_ × 9)]/Nt, where N_0_, N_1_, N_2_,… N_9_ = the number of plants with a canker score of 0, 1, 2, …9, respectively, and Nt =the total number of plants assessed. This information and a reference have now been added.

On page 13, authors mentioned a wide range in leaf petiole length and total leaf length but they had not included data for the leaf lamina length. It is important for the readers to know the range of leaf lamina length as if the length of the lamina is larger than that of the petiole then the fungus may not be able to reach the petiole, as the maximum distance travelled by the fungus in these investigations is merely 3cm in a susceptible line DY2. In this instance, it is obvious that there will be no correlation between total leaf and petiole length with the distance travelled by the fungus.

Answer: The range of leaf lamina length has been added as suggested.

I have noticed that authors inoculated the leaves very close to midrib, have they speculated the situation if lesions are far away from the midrib? How will that account for the fungus to reach to the petiole and into the stem?

Answer: To let Lm grow along the leaf petiole towards the stem, it is necessary to inoculate close to the midrib so that Lm can quickly reach the leaf petiole. In controlled conditions, inoculated leaves die quickly; if they are inoculated far away from the midrib, Lm may not reach the petiole before the inoculated leaf dies.

Fig. 3 need to be reordered from A to C, labels for Y and X-axis for each of the A, B and C need to be provided, though Y-axis label is common for A to C.

Answer: Done

Reviewer #2: This is a very well prepared manuscript. I only have a few comments.

Lines 132-134: It would be helpful if the previously-detected QTL within these DH lines and previouls field ratings (supporting their classification from susceptible to resistant) was listed, maybe in the Supplimentary?

Answer: The previously detected QTL and previous disease index in 7 years field experiments for the nine DH lines have now been provided as supplementary information (see S1 Table). The disease severity index shows that these DH lines range from susceptible to resistant.

Line 208 is missing a full stop between sentences.
Answer: Done.

Table 3 needs to be cleaned up a bit as the justification makes it hard to align numbers with rows/traits. As the figure is presented in Mb and the support intervals are also given in bp, perhaps the cM positions are expendible?

Answer: Due to the limited space available in portrait format, the Table 3 did not align up. We have now changed Table 3 to landscape and the rows now align up nicely. Due to the precision of genetic mapping, it is not useful to “extend” cM positions.

Lines 471-477: Later in the discussion you draw attention to previous work which tested QTL under controlled conditions using cotyledon infection. While the current study may be the first to do this with true leaf infection, the cotyledon assays still fall in the 'stage-1' of L. maculans growth, and should probably be mentioned here rather than later.

Answer: We have revised this paragraph and discussed the Lm growth stages later. Although the cotyledon assays still fall in 'stage-1' of Lm growth, cotyledon infection is not common in Europe where the main source of phoma stem canker is from leaf lesions that developed in autumn. The purpose of previous work on cotyledon assays done in Australia was for detection of *R* gene-mediated resistance (references 35 & 61) rather than for detection of QR. In the reference 61, since intermediate resistant phenotypes were observed on inoculated cotyledons, disease data were used for QTL detection.

6. PLOS authors have the option to publish the peer review history of their article ([what does this mean?](https://journals.plos.org/plosone/s/editorial-and-peer-review-process#loc-peer-review-history)). If published, this will include your full peer review and any attached files.

If you choose “no”, your identity will remain anonymous but your review may still be made public.

**Do you want your identity to be public for this peer review?** For information about this choice, including consent withdrawal, please see our [Privacy Policy](https://www.plos.org/privacy-policy).
Reviewer #1: No
Reviewer #2: No
